# Prioritizing the needs of children in a changing climate

**DOI:** 10.1371/journal.pmed.1002627

**Published:** 2018-07-31

**Authors:** Lawrence R. Stanberry, Madeleine C. Thomson, Wilmot James

**Affiliations:** 1 Department of Pediatrics, Columbia University Irving Medical Center, Columbia University, New York, New York, United States of America; 2 International Research Institute for Climate and Society, the Earth Institute, Columbia University, New York, New York, United States of America; 3 Department of Environmental Health Sciences, Mailman School of Public Health, Columbia University, New York, New York, United States of America; 4 School of International and Public Affairs, Columbia University, New York, New York, United States of America

## Abstract

In a Perspective, Lawrence Stanberry and colleagues discuss impacts of climate change on children.

Young children have no direct control over the environment they live in. They cannot control the quality of the air they breathe, the fluids they drink, the food they eat, or their exposure to contaminants or infectious diseases. Children are therefore far more vulnerable to climate-related disasters, and their care and protection from harm is more complicated. The onus is on adults to provide the protection and safety that children need. Here, we set out some specific challenges associated with the impacts of climate change on the world’s 2.3 billion children and suggest ways to address their underprioritized needs.

Anthropogenic climate change is predicted to increase the magnitude and frequency of extreme weather events (e.g., floods, droughts, and heat waves) that trigger humanitarian disasters or other emergencies [1] and that could exacerbate a wide range of environmental exposures that directly and indirectly affect children [2,3]. Children and adolescents are more vulnerable to these adverse exposures than adults because of a wide range of factors. They experience dynamic development and are different from adults anatomically, cognitively, immunologically, physiologically, and psychologically.

Changes in temperature may have a disproportionate impact on children’s well-being. Because of their greater body surface to volume ratio, infants and children are particularly vulnerable to dehydration and heat stress, and more heat-related deaths among infants are reported during heat waves [4]. Additionally, children are more likely to be affected by respiratory disease, renal disease, electrolyte imbalance, and fever during persistent episodes of hot weather [5]. Heat waves have also been shown to exacerbate the effects of allergens and air pollution (including particulates, nitrous oxide, and ozone) [6,7], which impact children more severely than adults because of their underdeveloped respiratory and immune systems and relatively high rates of respiration. As an example, observed reductions in wind speed associated with climate change are a factor in increasing pollution in Asian cities [8]. Similarly, studies of lung capacity suggest that the smog that has plagued Delhi, India in recent years have been particularly damaging to children [9].

Increasing temperatures may also expand the potential range of many vector-borne diseases. For example, the 2015 Zika virus epidemic has profoundly affected the lives of children and their families across Latin America and the Caribbean [10]. While research has substantially focused on severely affected microcephalic children, those who were asymptomatic at birth may develop problems later in life and should also be monitored clinically [11]. Extreme rainfall events are also projected to increase with climate change and may adversely affect the health of children beyond the immediate effects of drowning and crush injuries. For example, heavy monsoon rains, and subsequent flooding, in Pakistan in 2010 resulted in severe wasting of children as well as increased levels of diarrheal diseases, skin and eye infections, and leptospirosis; some children who lost their parents were forced to resort to begging, child labor, and prostitution [12]. In total, 2.8 million children under 5 years of age were affected, 1.4 million severely, and mortality increased from 94 to 110–120 deaths per 1,000 live births [13].

Studies suggest that climate change is increasing the intensity of North Atlantic hurricanes, thus increasing the likelihood that the severe consequences for children’s health will grow [14]. After hurricane Maria in 2017, medical responders in Puerto Rico encountered increases in gastroenteritis, asthma exacerbations, and skin infections (Arnab Ghosh, Cornell University, presentation at the Consortium of Universities of Global Health 2018 Annual Meeting; see Acknowledgments). Children were also at increased risk for mosquito-borne diseases such as the endemic Chikungunya and dengue, as well as leptospirosis through the drinking of contaminated water [15]. At around the same time, hurricane Harvey led to record rainfall over Texas, resulting in flood waters mixed with sewage and toxic chemicals [16]. The long-term implications for children’s health are unknown.

In some regions, severe droughts may in part be influenced by climate change, and in rural households, there may be significant impacts on child well-being and development through increased food insecurity and dietary changes [17]. Droughts may also have destabilizing effects on society, contributing to conflict and forced migration in resource-poor settings and thereby increasing children’s vulnerability to a wide range of health issues [18]. Similarly, children’s access to basic healthcare services may be uncertain. In extreme situations, there may be risks of forced child labor, sexual exploitation, violence, abuse, child marriage, and incarceration.

In response to climate-related disasters, specific protocols are needed for triage, decontamination, and care for children exposed to climate-induced hazards, whether physical, biological, or chemical. The needs of children must also be factored into medical commodity supply chains. Children require appropriate medical supplies and hospital equipment such as endotracheal tubes and hospital cribs. Drug metabolism and pharmacokinetics are different in children, and treatment dosages cannot be extrapolated from adult data but must be determined by specific studies. During emergencies, oral treatments may not be available in liquid formulations, and most children under six years of age cannot swallow pills. Children have smaller blood volumes and may pose challenges as regards drawing blood for diagnostic testing. To meet their needs, serologic tests should have good performance characteristics and use a very minimal amount of blood, ideally the amount that can be collected by capillary sampling.

Children and adolescents are also more susceptible to traumatic events that can result in long-term negative effects on health, social, and behavioral outcomes. Mental health services must be part of the disaster planning to meet the needs of children, as indicated by the events in Orissa, India following the “super cyclone” of 1999 [19]. However, children and adolescents should not only be seen as victims of climate-related catastrophes; as stated in the Sendai Framework for disaster risk reduction, they are also agents of change, bringing new energy and knowledge to their families and communities [20]. The health co-benefits to children of cleaner air are now well understood [6] and have become a rallying cry for climate change action.

To begin to address the needs of children and their families when confronted with climate-change–related health disasters, we propose three considerations. First, an international consortium of experts should be established, ideally by WHO and UNICEF, to develop adoptable medical and behavioral protocols and to set research agendas to identify and address the unmet specific needs of children. Second, the development of best practice guidelines for climate-change–related event planning is required incorporating strategies for addressing the health and care of children. Finally, funding mechanisms that are designed to help the most vulnerable countries prepare for and respond to climate-related disasters are required, and these mechanisms must consider the development of responses that specifically address the unmet needs of children’s health.
